# Assessment of Knowledge, Attitudes, and Practices towards Rift Valley Fever among Livestock Farmers in Selected Districts of Malawi

**DOI:** 10.3390/tropicalmed7080167

**Published:** 2022-08-05

**Authors:** Henson Kainga, James Mponela, Linda Basikolo, Marvin Collen Phonera, Prudence Mpundu, Muso Munyeme, Edgar Simulundu, Ngonda Saasa

**Affiliations:** 1 Department of Veterinary Epidemiology and Public Health, Faculty of Veterinary Medicine, Lilongwe University of Agriculture and Natural Resources, Lilongwe 207203, Malawi; 2 Department of Disease Control, School of Veterinary Medicine, University of Zambia, Lusaka 10101, Zambia; 3 Department of Animal Health and Livestock Development, Ministry of Agriculture, Lilongwe 207203, Malawi; 4 Department of Environmental and Occupational Health, Levy Mwanawasa Medical University, Lusaka 33991, Zambia; 5 Macha Research Trust, Choma 20100, Zambia

**Keywords:** attitude, knowledge, Malawi, practice, predictors, Rift Valley fever

## Abstract

Rift Valley fever (RVF) is a mosquito-borne viral zoonosis whose cases go unreported in endemic areas without active surveillance. Information on the knowledge, attitude, and practice of RVF among livestock farmers remains speculative in Malawi. A cross-section survey using a semi-structured questionnaire (*n* = 400) was conducted in eight districts of Malawi to capture information on knowledge, attitude, and management practices (KAP) regarding RVF. The average KAP score was calculated from total scores for knowledge, attitude, and practices and then assessed. The association between the level of knowledge and factors of knowledge, factors of attitude, and factors of practices was determined using Pearson chi-square. Multivariate analysis was used to determine the predictors of knowledge. Participants had an overall poor knowledge (score = 17.94%), negative attitude (score = 9.40%), and poor management practices (score = 41.23%) towards RVF. Only 8.25% (33/400) of participants had sufficient knowledge of RVF. The study found that the cause of abortion (OR: 3.86 (95% CI: 1.14–13.05)) (*p* = 0.030) and knowledge on transmission of RVFV (OR: 5.65 (95% CI: 1.76–18.12)) (*p* = 0.004) were predictors of insufficient knowledge of RVF. The current study reported that participants had insufficient knowledge and a negative attitude despite displaying limited management practices towards RVF. Therefore, this study recommends community sensitization to RVF and advocates for the importance of reporting suspected cases to relevant authorities for proper management.

## 1. Introduction

Rift valley fever (RVF) is a per-acute or acute zoonotic disease of ruminants that is endemic in Africa. The disease is caused by a single serotype of a mosquito-borne bunyavirus of the genus *Phlebovirus,* a member of *Phenuiviridae* viridae, namely the Rift Valley fever virus (RVFV) [1]. Infection in animals is highly associated with the presence of *the Aedes McIntosh* mosquito vector [2]. The transmission of RVFV to livestock is via some species of mosquito such as *Culex*, *Anopheles*, and *Aedine* [2,3], which are widely distributed in the southern region of Africa [2,3,4].

Older non-pregnant animals, although susceptible to infection, are more resistant to clinical disease [5]. Within ruminant hosts, RVF is more severe in sheep than goats and cattle, where it causes abortion storms and high mortality in neonates [6]. RVF in humans is predominantly linked to contact with infected animals and animal body fluids and is characterized by a mild influenza-like disease that is sometimes self-limiting. However, in some cases, it may cause blindness, abortions, and even deaths [7,8].

In humans, RVF usually causes mild to acute undifferentiated fever, but in severe cases, hemorrhagic fever, neurological disorders, or blindness and death can occur [9]. Apart from causing human disability and suffering and livestock disease, RVFV also causes economic losses arising from measures restricting movement, slaughter, and exports of livestock and livestock products [10,11,12]. The most severe outbreaks during the 1997–1998 and 2006–2007 seasons caused 478 and 309 human deaths, respectively, in Tanzania, Kenya, and Somalia [10,13,14,15]. According to Sindato et al. [13], the 2007 outbreak in Tanzania had a severe impact on the international animal trade, in which there was a 54% decline in exports equivalent to a loss of $352,750.00. The estimated loss as a result of cattle mortalities was $4,243,250.00, whereas that of goats and sheep was $2,202,467.00.

However, it is challenging to establish the true extent of losses attributable to RVF during the interepidemic period (IEP), as most cases are unconfirmed due to a lack of diagnostic capacity [15,16,17]. Most of the cases that share clinical signs with RVF in the field are occasionally presumed to be East Coast Fever (ECF) and Brucellosis [18,19], despite testing negative. The unconfirmed cases of RVF could account for a greater proportion of the overall losses arising from abortion and other adverse events. Consequently, the lack of empirical epidemiological data on RVF poses a challenge to quantifying the impact of control measures [20].

RVF is endemic in sub-Saharan Africa, and the virus has crossed the African boundaries to Mayotte, France [21]. RVF has been reported in all neighboring countries of Malawi, namely Mozambique, Tanzania, and Zambia, within the last seven years [22,23,24]. The last report of RVF in Malawi was in 1992 [25]. Since then, Malawi has not reported on the presence of RVF despite the occurrence of probable cases in livestock and humans. Malawi experienced a suspected RVF outbreak (2006–2007) among smallholder dairy herds of Thyolo and Chiradzulu districts. The disease caused massive cases of abortion among dairy cattle and goats in the southern region, mostly within the milk catchment area of the Shire Highlands Milk Producers Association (SHMPA). Epidemiologically, the disease appears to have been sparked by the movement of animals from RVF-infected regions of Tanzania, where the animals were sourced by World Vision International Malawi (WVI) (GoM, 2008, unpublished). The laboratory test results were negative for ECF and Brucellosis. Regrettably, the laboratory tests did not confirm suspected RVF cases due to a lack of diagnostic capacity. To generate a plausible understanding of the prevailing epidemiology of RVF in Malawi, there was a need to engage livestock farmers through a participatory epidemiological approach [26,27].

Further, regardless of numerous probable cases of RVF, the livestock farmers do not consider the possible circulation of RVFV and the risk of transmission of the disease among livestock and humans. Based on this background, it was necessary to conduct a study of knowledge, attitudes, and practice (KAP) to collect information and identify knowledge gaps, behavioral patterns, and management practices that could influence suspecting and reporting probable RVF cases [15,28]. The gathered information was intended to facilitate designing further RVFV investigations in livestock. In this study, we aimed to report knowledge, attitudes, and management practices regarding RVF among livestock farmers that could account for the failure to report or underreporting of susceptible cases in livestock herds.

## 2. Materials and Methods

### 2.1. Study Site

**Figure 1:**
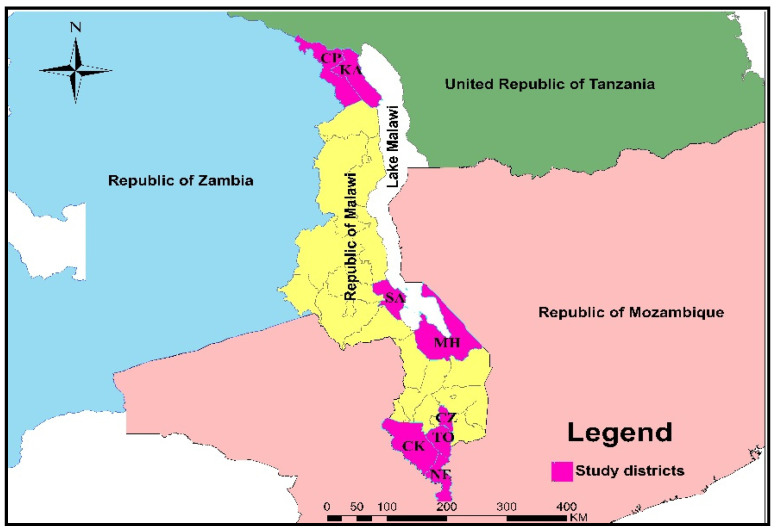
Map of Malawi showing the study districts. CP = Chitipa, KA = Karonga, SA = Salima, MH = Mangochi, CZ = Chirazulu, TO = Thyolo, CK = Chikwawa, and NE = Nsanje.

Malawi is a landlocked country in sub-Saharan Africa, situated between latitudes 9° and 18° S and longitudes 32° to 36° E. It shares borders with Tanzania to the north, Mozambique to the southeast and southwest, and Zambia to the west. Eight districts, namely, Chitipa (CP), Karonga (KA), Salima (SA), Mangochi (MH), Chiradzulu (CZ), Thyolo (TO), Chikwawa (CK), and Nsanje (NE), were purposively selected due to their high livestock density, high rainfall intensity, flooding, cross-border livestock movement, and vegetation cover (Figure 1). CP and KA districts are located in the northern region of Malawi, bordering Tanzania and Zambia. SA (central region) and MH (southern region) are located along the shores of Lake Malawi with a characteristic wide range of dambo areas and vegetation cover; TO and CZ (southern) are located adjacent to the Zomba district, where RVF was previously reported [25,29]. CK and NE districts are located in the southernmost part of Malawi in the Shire valley and along the border with Mozambique. The area has game reserves and many irrigation schemes.

### 2.2. Sample Size

A cross-sectional survey was conducted among the livestock farmers in eight districts. An exploratory research method was used to estimate the sample size because there is a paucity of literature reviews on knowledge and predictors of RVF in Malawi. The researcher used Thumb’s rule of 30% in determining the sample size, which was calculated using Cochran’s [30]:

$$n_{0}=\frac{Z^{2}\mathrm{pq}}{e^{2}}\;=384$$

where **n** = required sample size, Z = 1.96 (confidence level at 95%), p = prevalence rate of the RVF virus (50% estimated because prevalence was unknown in the study area), q = 1 − p, e = level of precision at 5% (standard value of 0.05)

The investigators considered a 10% attrition rate as previously described by Adegoke et al. [31].

(1) $$\mathrm{Attrition}=\frac{\mathrm{calculated}\;\mathrm{samples}\;\mathrm{size}\;\times \;\mathrm{Attrition}\;\mathrm{rate}}{\mathrm{Attrition}\;\mathrm{rate}\;-1\;\mathrm{attrition}}=\frac{384\times 10\;}{10-1}=\frac{3840}{9}=426$$

attrition = 426 − 384 = 42. Therefore, new sample size = 384 + 42 = 426.

### 2.3. Data Collection Instruments

A structured questionnaire was adapted from the Focus Group Discussion (FGD) guide, which was conducted earlier and further developed through an in-depth literature search that was translated and pretested in the Chichewa language. The questionnaires were pre-validated for relevance, accuracy, clarity, simplicity, and understandability, and the Cronbach`s alpha coefficient was 0.72, 0.86, and 0.78 for the questionnaire for knowledge, attitude, and practices, respectively, indicating the internal consistency and reliability of the study instrument. A pilot study of the questionnaire was performed on 16 participants that were excluded from the final analysis. Further, adequate training was provided to enumerators to clear the observed discrepancies and improve the quality of the collected information. The training was conducted at the Department of Epidemiology and Public Health, Faculty of Veterinary Medicine, Lilongwe University of Agriculture and Natural Resources (LUANAR).

### 2.4. Participant Identification and Data Collection

A structured questionnaire with mostly categorical questions to ease data processing and improve the precision of responses was administered in the Chichewa language. Only smallholder farmers of cattle, goats, and sheep were included in the study. The study strictly considered participants above 18 years of age to include those responsible for keeping animal health records. At each District Agriculture Office, 10% of the recorded livestock farmers were considered using a simple random sampling method. Randomly selected livestock farmers were asked to complete a written consent form. The farmers that declined to give consent were replaced. Eventually, the questionnaire to capture knowledge, attitude, and management practices information on RVF was administered face to face to 400 smallholder livestock owners. Herd size was categorized into small herds (<24) and large herds (≥25) [32]. The distribution of participants per district was 39 CP, 45 KA, 60 SA, 54 MA, 36 TO, 48 CZ, 52 CK, and 66 NE. Data were collected in the period of May–June 2020.

### 2.5. Determining KAP Scores

Thematic questions regarding expected knowledge, management practices, and attitudes regarding RVF were answered on a “yes” or “no” basis. A correct answer was assigned 1 point, while an incorrect or “don’t know” answer was assigned 0 points. The total knowledge score ranged from 0 to 100%, with a higher score denoting a better knowledge of RVF. To determine the KAP score level, the cut-off value was based on the ability of the participant to explain the cause, host range, clinical signs, and mode of transmission of RVF. An average KAP score of 80% or more was classified as “good knowledge”, indicating satisfactory knowledge to suspect and report any probable case of RVF. On the other hand, an average KAP scale of less than or equal to 79% was classified as “poor knowledge” indicating unsatisfactory knowledge. A similar scoring approach was used to classify “positive attitude”, “negative attitude”, “good practice”, and “poor practice”, as adapted from [33,34].

### 2.6. Data Processing and Analysis

Data were entered, cleaned, and validated in Microsoft Office Excel^®^2019. Data entry and analysis were consistently cross-checked by supervisors. The data were grouped according to intended information as socio-demographic, knowledge, attitude, and management practices. Data analysis was conducted using SPSS Ver. 21 (IBM Corp, Armonk, NY, USA) statistical software and split into three stages: first, univariate analysis for descriptive statistics such as percentages and frequencies of each independent variable, followed by a bivariate analysis to assess the association between each independent variable and the dependent variables, with only potential predictors with a *p*-value less than 0.25 being considered for the multivariate analysis. Prior to multivariate analysis, three univariable linear regression models with the observed level of knowledge were generated for the categories of knowledge, attitude, and practice. The univariable linear regression model was fitted with all significant independent variables for each category to check for multicollinearity. Multicollinearity was checked by Variance Inflation Factors (VIF) (VIF value < 1.00) and Tolerance (value > 0.20). Thereafter, a multivariable linear regression model was fitted, which included variables that retained significance (*p* < 0.05) upon univariable analysis [35,36]. For the multivariate analysis, the stepwise regression method and enter algorithms were used, then independent variables with a *p*-value less than 0.05 were considered significant predictors of RVF knowledge. The generated model was tested for goodness of fit and predictability using the Hosmer–Lemeshow test and the Omnibus test, respectively.

### 2.7. Ethics Statement

Animal Health Committee of the Department of Animal Health and Livestock Development (DAHLD-AHC: Ref. DAHLD/AHC/10/2019), Malawi and University of Zambia Biomedical Research Ethical Committee (UNZABREC: Ref. 617-2019), Zambia, independently reviewed and approved the research protocol.

## 3. Results

### 3.1. Socio-Demographic Characteristics

Of a total of 400 participants enrolled in the study (Table 1), 67.25% (269/400) were males, while 32.75% (131/400) were females. The age group of 46 years and above were in the majority (39.75%, 159/400), followed by the age group of 36–45 (35.50%, 142/400). The majority of the participants (67.50%, 270/400) had primary education, and few (0.75%, 3/400) had attained tertiary education.

#### 3.1.1. Knowledge of Participants of the RVF and KAP Score

Of the participants, 10.25% (41/400) knew RVF, although only 8.25% (33/400) knew its causative agent and 8.75% (35/400) its clinical signs (Table 2). The host species of RVF were mentioned by 9.50% (38/400) of the participants. Further, only 9.50% (38/400) of the participants knew its transmission pattern, and only 9.50% (38/400) believed that the mosquitoes could transmit RVFV. The majority of the participants (92.50%, 370/400) witnessed abortion in livestock. The study also found that 9.50% (38/400) of the participants knew that RVF is zoonotic. The average KAP score of RVF knowledge of the participants was found to be 17.94% (143.5/8), which indicated an unsatisfactory level with regard to the ability to suspect and report probable cases of RVF.

#### 3.1.2. Management Practices towards RVF and KAP Score

Of the participants, 84.75% (339/400) witnessed neonatal death in livestock. About 44.75% (179/400) of the participants handled the aborted materials with unprotected hands, and 26.75% (107/400) of the participants handled neonatal death materials with unprotected hands. Over half of the participants (55.50%, 222/400) did not bury the aborted materials. Nevertheless, 9.50% (38/400) of the participants reported being capable of suspecting RVF in livestock. On the other hand, only 9.50% (38/400) indicated being capable of preventing the spread of RVF among livestock (Table 3). The average KAP score for RVF management practices of participants was found to be 41.23% (375.1/9), which indicated an unsatisfactory level with regard to the ability to suspect and report probable cases of RVF.

#### 3.1.3. Attitude of Participants towards RVF and KAP Score

Of the participants, 84.25% (337/400) associated heavy rainfall and flooding with the destruction of crops and homes, while 15.75% (63/400) of the participants associated heavy rainfall and flooding with the spread of RVF. Further, 8.50% (34/400) of the participants associated increased mosquito population with the spread of RVFV. Furthermore, 91.50% (366/400) of the participants did not associate abortion and neonatal death with the possible presence of RVF. Only a few participants (8.50%, 34/400) could associate production losses with the possibility of RVF infection. In addition, 8.50% (34/400) of participants did not fear suffering RVF (Table 4). The average KAP score of RVF attitude among participants was found to be 9.40% (75.2/8), which indicated an unsatisfactory level with regard to the ability to suspect and report probable RVF cases.

#### 3.1.4. Mean Knowledge, Attitude, and Practices across the Socio-Demographic Characteristics

The study found that men had higher mean scores of 63.50 ± 17.90, 24.88 ± 8.13, and 147.44 ± 72.52 than females for knowledge, attitudes, and practices, at *p* = 0.019, *p* = 0.014, and *p* = 0.003, respectively. Additionally, mean scores statistically differed among marital status groups and varied significantly with herd size categories (Table 5). Mean knowledge scores were higher 25.13 ± 3.52 and 16 ± 11.51 for Thyolo and Chiradzulu districts, respectively, than for other districts. The age group greater than 46 had a higher mean score of 37.62 ± 15.63, followed by the 36–45 age group, with a mean score of 21.87 ± 15.04.

### 3.2. Analysis of the Association between RVF Knowledge of Participants and Potential Predictors of Knowledge

The study that found only 8.25% (33/400) of participants were knowledgeable about RVF at a cutoff point of 80% and above. Pearson chi-square was run to assess the association between the potential predictors and the knowledge. There was an association between knowledge and the observed level of knowledge on what causes RVF (*X*^2^ = 7.989, *p* = 0.018); the observed level of knowledge on clinical signs (*X*^2^ = 4.007, *p* = 0.045); and the observed level of knowledge on the mode of transmission (*X*^2^ = 13.214, *p* = 0.001) under the knowledge category. Additionally, there was an association between the knowledge and the lack of the ability to prevent the spread of RVF (*X*^2^ = 18.105, *p* = 0.001) and the practice of mixing young and old livestock (*X*^2^ = 14.192, *p* = 0.001) under the management practices. There was no association between the knowledge and potential predictors within the negative attitude category. Thereafter, the variables were screened for multicollinearity using univariate linear regression (Table 6) and (Table 7).

### 3.3. Predictors of RVF Knowledge for the Participants

After the adjustment for other variables in the stepwise binary logistic regression model, significant predictors of RVF knowledge for participants were with a *p*-value < 0.05. Variables with a *p*-value < 0.250 in the bivariate analysis were included in the model. The test had an insignificant Hosmer–Lemeshow goodness-of-fit statistic (*p* = 0.828), and the Omnibus Test of Model Coefficients values of *p* < 0.000 were obtained, indicating the goodness of fit of the generated model. The significant predictors were the knowledge of the cause of abortion and knowledge of how RVFV is transmitted, and the respective adjusted odds ratios (aORs) are presented in Table 8. Farmers with the knowledge that misfortune caused the abortion were aOR: 3.861 (95% CI: 1.14–13.05) times more likely to be less knowledgeable about RVF than farmers with knowledge of diseases that cause abortion (*p* = 0.001). Farmers without knowledge of how RVFV is spread/transmitted were aOR: 5.65 (95% CI: 1.76–18.12) times more likely to be less knowledgeable about RVF than farmers with knowledge of how RVFV is spread/transmitted (*p* = 0.004). Farmers with knowledge of the affected species were (aOR: 0.140 (95% CI: 0.03–0.62) times more likely to be knowledgeable about RVF than farmers without knowledge of the affected species (*p* = 0.009).

## 4. Discussion

Rift Valley fever is a re-emerging mosquito-borne viral hemorrhagic fever in Africa and the Arabian Peninsula, affecting humans and livestock. The current study revealed poor knowledge, negative attitude, and poor management practices that were not satisfactory to suspect and report probable RVF cases. Consequently, this compromises the prevention and control efforts as previously reported by [15].

Good knowledge, attitudes, and practices regarding RVF amongst livestock farmers form a great foundation for infection control and prevention in areas at risk for RVF. This study aimed to assess livestock farmers’ knowledge, attitudes, and practices because of the critical role that knowledge plays in curbing the spread of viral hemorrhagic infectious diseases, including RVF [37]. In addition, the understanding of the influence of management practices and attitudes toward infectious diseases is crucial to improving prevention and control efforts.

The demographic information of the respondents included marital status, education, gender, and age group. Gender and age are among the important demographic factors that could contribute to the knowledge, attitude, and practices of farmers [38,39]. By integrating gender and age in a sampling of the respondents, the response bias could be reduced, and the findings could be easily generalized. Education opens the way for awareness and fosters a better understanding of conditions and topics in the production community. The positive influence of education was noted based on the mean score of secondary and tertiary education, as was observed in other studies [40,41]. The mean scores for knowledge, attitudes, and practices of married farmers were higher compared to other categories (Table 5) because livestock management was provided by family members. Family members endeavor to share information in order to achieve increased benefits from their farming in agreement with reports [32,42,43].

This study established that few farmers knew about RVF, despite having unspecified sources of information (Table 2 and Table 5). This level of awareness is believed to result from a previously suspected RVF outbreak (2006–2007) observed among smallholder dairy herds of Thyolo and Chiradzulu districts. Smallholder dairy farmers operate in Milk Bulking Groups (MBG), where they receive expert training on various livestock topics, including diseases, and share information and experiences among themselves. Certainly, MBG gatherings promote the awareness of RVF among livestock farmers. Nevertheless, the farmers had limited knowledge of RVF, its causative agents, transmission, clinical signs, and host species, which are central pillars for describing the disease, as previously reported by [36,44]. The observed basic and clinical knowledge of RVF was insufficient to enable farmers to suspect and report probable cases of RVF in livestock, as previously reported [15,44]. The current study found that farmers did not know that RVF was a zoonotic infectious disease transmitted between animals and humans. Ng’ang’a et al. [45] reported a lack of knowledge of zoonotic for RVF among pastoral communities in northern Kenya, a finding that is in agreement with the present study. The unsatisfactory knowledge of RVF as a zoonotic disease could be attributable to a lack of zoonotic knowledge among livestock farmers. This observation corroborated the results on management practices whereby participants handle aborted materials and remains of neonatal death without protection. These materials are potential risk factors for RVFV seropositivity in humans and livestock, as reported by [46].

The poor knowledge among farmers of the role of mosquitoes in the transmission of the disease draws particular concern, as only 9.5% of the participants knew that the mosquito is the primary vector for transmission of RVF in livestock (Table 2). Further, farmers did not know that humans can contract RVF infection through mosquito bites or coming into contact with infectious aborted fetuses and other remains of neonatal death materials. Furthermore, participants were not aware that RVF is zoonotic, a threat to human health, which could be due to poor awareness. Many emerging diseases are zoonotic infectious diseases transmitted between animals and humans; examples include RVF [47]. These observations show that farmers do not practice preventive measures against these risk factors, as previously reported [36].

The poor management of potentially infectious materials was common, as over half of participants (55.50%) did not bury aborted materials, and 44.80% handled the aborted materials with unprotected hands, which were practices of particular concern regarding RVF disease control and prevention. The possible risk of RVFV transmission in the study population could be assumed to be high, considering deficiencies in practices of the management of aborted materials, dystocia, and the remains of neonatal death that were handled without protective equipment and sometimes disposed of incorrectly, as alluded to by [28,43]. This malpractice is common in areas where farmers have low knowledge of RVF, as was the case in Ijala district, Kenya [47]. The authors strongly encourage responsible authorities to embark on the community sensitization of proper handling and disposal of high-risk materials such as aborted fetuses and neonatal death materials.

Although not satisfactory, the participants had a reasonable management practice score (Table 3). The average KAP score (41.23%) showed that the participants had the potential to improve with enhanced awareness. Most of the management practices for RVF are similar with respect to production practices and the prevention of other diseases. It has been observed that farmers in participating districts allowed livestock to graze in communal grazing grounds with mixed-livestock species. Communal grazing and mixed-species grazing accounted for the intensive interaction of livestock herds between different villages. These interactions could potentially influence the spread of RVFV between livestock and possible spillover into humans [48].

The observed attitudes of participants could be considered a mediator between knowledge and practices and have a significant role in directing the choice of management practice. Attitudes support the process of changing individuals’ behavior [49]. This was evident by the findings whereby cases of dystocia, abortion, and neonatal death in livestock were perceived as a result of other diseases, misfortune, or poor feeding (starvation and poisonous plants), which is similar to the findings reported by [19,50]. In addition, few farmers admitted losses caused by suspected cases of RVF on their farms. The failure to associate the cause of losses in livestock indicated gross negligence, and there is no potential to suspect and report possible cases of RVF. Most farmers could not suspect RVF because they did not know the disease by name or provide a vernacular name and could not differentiate it from other diseases with similar clinical signs such as brucellosis and bovine viral diarrhea (BVD) [51,52]. This suggests that many diseases with similar clinical signs such as RVF exist but have not been confirmed or described. The foregoing information suggests that the occurrence of abortions could be attributed to other causes, including RVFV. The poor attitudes of farmers regarding RVF were observed in the failure to associate the increased mosquito population, heavy rainfall, and flooding with increased abortions during the period of January, February, and March, as opposed to the frequency of abortions in April, May, and June. Nevertheless, the attitude and the level of knowledge of livestock farmers regarding RVF were not effective in suspecting or reporting probable cases of RVF [53] and were evident in the attitude and management practices of the majority of the farmers in the study area [15].

Table 5 shows that knowledge was observed in two districts, Thyolo and Chiradzulu, which would suggest that there were sporadic cases similar to the previous experience of suspected RVF cases (2006–2007) that sparked memory and the sharing of information, as previously reported [36]. Farmers with secondary and tertiary education coupled with an age of greater than 45 years had better knowledge and attitude and subsequently better practices. These observations may be because education supports informed decisions, while experience accounts for the degree of familiarity with the presentations of the disease. Farmers with fewer than 25 livestock had better knowledge, attitude, and practices than farmers with more than 25 livestock, as their main source of labor was family members. However, both groups of farmers (with <25 livestock and with ≥25 livestock) indicated that they cannot prevent the disease, maybe because the prevention of mosquitos is likely to be cumbersome compared to the control of other external parasites such as ticks. Further, having knowledge does not directly translate into the capacity to prevent RVF, as previously reported [53].

All viral hemorrhagic infectious diseases, including RVF, require special attention in prevention and control [54]. This study reported associations between knowledge and observations under management practices and attitude categories that synergistically influenced negative attitudes of farmers with respect to conducting good practices to prevent RVF. For instance, the association between the observed low knowledge with knowledge of clinical signs and causative agents of RVF strongly indicated that poor knowledge corroborated the failure of farmers to find out the cause and clinical signs of RVF (Table 6) [55]. Another association was observed between low knowledge and the ability to prevent RVF through practice, which is of particular concern (Table 7). This association indicated the dependence between the low knowledge of RVF and the inability to prevent it [53]. Additionally, there was an association between low knowledge and the three observations regarding attitudes: that RVF is not a cause of neonatal death, that RVF is not a cause of production losses, and a lack of fear of suffering from RVF. This association could indicate low awareness of the disease and consequently limited efforts made towards the detection of the disease.

Nevertheless, the study found two factors that influenced the low knowledge of RVF among farmers (Table 8): a lack of knowledge of the cause of abortion (misfortune) and a lack of knowledge of the transmission of RVFV. Misfortune in this context blatantly obscures the inquisitive minds on the causes of abortion. On the other hand, poor feeding was not the best option to consider, as the study was conducted in the wet season when green grasses were in abundance. In addition, a lack of knowledge of how RVFV is transmitted and the role of mosquitoes in the transmission of RVFV predisposes the farmers to the spread of RVFV among the livestock and/or spill it over into humans [47]. These behaviors could be influenced by a lack of knowledge among the farmers and may not prompt the communities to suspect and report probable cases of RVF.

The study revealed the possible associations between low knowledge and negative attitudes, poor knowledge, and poor management practices, showing that improving knowledge levels of farmers would subsequently improve attitude and practice, hence fostering the capacity to suspect and report probable cases of RVF.

## 5. Conclusions

This study has revealed that respondents had low knowledge, negative attitudes, and poor practices regarding RVF. The effective prevention and control of RVF require improved awareness among farmers about the disease in livestock. Therefore, this study recommends community sensitization to RVF and advocates for the importance of reporting suspected cases to relevant authorities for proper management. The study suggests the investigation of RVFV sero-prevalence in humans working in risky areas.

## Figures and Tables

**Table 1:** Summary of socio-demographic characteristics.

Variable	Category	Frequency	Proportion (%) *n* = 400	95% CI
Gender				
	Male	269	67.25	62.38–71.78
	Female	131	32.75	28.21–37.62
Age (years)				
	18–25	44	11.00	8.19–14.58
	26–35	55	13.75	10.38–17.33
	36–45	142	35.50	30.85–40.43
	≥46	159	39.75	34.95–44.75
Education				
	None	42	10.50	7.75–14.03
	Primary	270	67.50	62.63–72.02
	Secondary	85	21.25	17.41–25.65
	Tertiary	3	0.75	0.19–2.36
Marital Status				
	Married	352	88.00	84.31–90.93
	Single	18	4.50	2.77–7.15
	Divorced	24	6.00	3.96–8.92
	Widowed	6	1.50	0.16–3.40
Herd size				
	<25	379	94.75	91.96–96.64
	≥25	21	5.25	3.36–8.04
District				
	CP	39	9.75	7.11–13.19
	KA	45	11.25	8.40–14.86
	SA	60	15.00	11.72–18.97
	MH	54	13.50	10.39–17.33
	CZ	36	9.00	6.47–12.35
	TO	48	12.00	9.06–15.69
	CK	52	13.00	9.94–16.79
	NE	66	16.50	13.07–20.57
Species on the farm				
	Cattle	35	8.75	6.25–12.07
	Cattle, goat	187	46.75	41.79–51.77
	Cattle, goat, sheep	106	26.50	22.30–31.16
	Goat	48	12.00	9.06–15.69
	Goat, sheep	21	5.25	3.36–8.04
	Sheep	3	0.75	0.19–2.36

*n* = Number of participants; CI = 95% Confidence Interval.

**Table 2:** Participants’ knowledge of Rift Valley fever.

Factors of Knowledge	Category	Frequency (*n* = 400)	Proportion (%)	95% CI	KAP Score (%)
Did your livestock abort					
	Yes	370	92.50	89.35–94.80	
	No	30	7.50	5.19–10.64	
Which months					
	Jan, Feb, Mar	349	87.25	83.86–90.27	
	Apr, May, Jun	51	12.75	9.72–16.51	
What causes abortion					
	Diseases	313	78.25 *	73.81–82.12	78.25
	Poor feeding	48	12.00	9.06–15.69	
	Misfortune	39	9.75	7.10–13.19	
Do you know RVF					
	Yes	41	10.25 *	7.54–13.75	10.25
	No	359	89.75	86.25–92.46	
Do you know clinical signs of RVF?					
	Yes	35	8.75 *	6.25–12.07	8.75
	No	365	91.25	87.93–93.75	
Do you know what causes RVF					
	Yes	33	8.25 *	5.83–11.50	8.25
	No	367	91.75	88.49–94.17	
Do you know RVF host species					
	Yes	38	9.50 *	6.89–12.91	9.50
	No	362	90.50	87.09–93.11	
Do you know how it is transmitted					
	Yes	38	9.50 *	6.89–12.91	9.50
	No	362	90.50	87.09–93.11	
Can mosquito transmit RVF					
	Yes	38	9.50 *	6.89–12.91	9.50
	No	362	90.50	87.09–93.11	
Do you know that it is zoonotic					
	Yes	38	9.50 *	6.89–12.91	9.50
	No	362	90.50	87.09–93.11	
Average KAP score on RVF knowledge of participants (143.5/8)					17.90%

*n* = number of participants; * = Proportion considered as KAP score; CI = Confidence interval.

**Table 3:** Participants’ practice and management of livestock in terms of Rift Valley fever.

Factors under Management Practices	Category	Frequency (*n* = 400)	Proportion (%)	95% CI	KAP Score (%)
Did you experience neonatal death					
	Yes	339	84.75	80.76–88.05	
	No	61	15.25	11.95–19.24	
Did you experience retain placenta					
	Yes	228	57.00	51.98–61.88	
	No	172	43.00	38.11–48.02	
Are young and old livestock raised together					
	Yes	233	58.25 *	53.23–63.10	58.25
	No	167	41.75	36.89–46.76	
How did you handle aborted materials					
	Protected hands	221	55.25 *	50.22–60.17	55.25
	Unprotected hands	179	44.75	39.83–49.77	
How did you dispose aborted materials					
	Buried	178	44.50 *	39.58–49.52	44.50
	Unburied	222	55.50	50.47–60.42	
How did you handle neonatal death materials					
	Protected hands	293	73.25 *	68.58–77.47	73.25
	Unprotected hands	107	26.75	22.53–31.42	
Can you suspect RVF in livestock					
	Yes	38	9.50 *	6.89–12.91	9.50
	No	362	90.50	87.09–93.11	
Can you prevent RVF in livestock					
	Yes	38	9.50 *	6.89–12.91	9.50
	No	362	90.50	87.09–93.11	
Mode of night shelter					
	Communal	177	44.25	39.34–49.27	54.00
	Private	216	54.00 *	48.97–58.94	
	None	7	1.75	0.77–3.73	
Type of grazing grounds					
	Communal	224	56.00	50.97–60.90	44.00
	Private	176	44.00 *	39.09–49.02	
Type of herd composition					
	Mixed species	293	73.25	68.57–77.46	26.75
	Single species	107	26.75 *	22.53–31.42	
Average KAP score on RVF attitude of participants (375.1/9)					41.23%

*n* = number of participants; * = Proportion considered as KAP score; CI = 95% Confidence interval.

**Table 4:** Attitudes of participants towards Rift Valley fever.

Factors under Attitude	Category	Frequency (*n* = 400)	Proportion (%)	95% CI	KAP Score (%)
How do you feel on heavy rainfall and flooding?					
	Destroy crops	337	84.25	80.22–87.60	
	Promote spread of RVF	63	15.75 *	12.39–19.78	15.75
How do you feel on increased mosquito population, can it spread RVF?					
	Yes	34	8.50 *	6.04–11.78	8.50
	No	366	91.50	88.21–93.95	
Do you think RVF cause abortion?					
	Yes	34	8.50 *	6.04–11.78	8.50
	No	366	91.50	88.21–93.95	
Do you think RVF cause neonatal death?					
	Yes	34	8.50 *	6.04–11.78	8.50
	No	366	91.50	88.21–93.95	
Do you think there is production losses on your farm due to RVF?					
	Yes	34	8.50 *	6.04–11.78	8.50
	No	366	91.5	88.21–93.95	
Do you think vendors bring RVF infected livestock?					
	Yes	34	8.50 *	6.04–11.78	8.50
	No	366	91.50	88.21–93.95	
Do you fear suffering RVF?					
	Yes	34	8.50 *	6.04–11.78	8.50
	No	366	91.50	88.21–93.95	
Are you capable to prevent RVF in livestock?					
	Yes	34	8.50 *	6.04–11.78	8.50
	No	366	91.50	88.21–93.95	
Average KAP score on RVF attitude of participants (75.2/8)					9.40%

*n* = number of participants; * = Proportion considered as KAP score; CI = Confidence interval.

**Table 5:** Mean knowledge, attitude, and practice scores across socio-demographic characteristics.

Variable	Mean Knowledge Score		Mean Attitude Score		Mean Practice Score	
	Mean	Std. Deviation	Mean	Std. Deviation	Mean	Std. Deviation
**Gender**						
Male	63.50	17.90	24.88	8.13	147.44	72.52
Female	8.25	4.82	12.75	2.12	45.22	36.86
*p*-value	**0.019**		**0.048**		**0.003**	
**Age (years)**						
18–25	3.50	4.24	1.38	1.06	23.89	15.35
26–35	6.25	5.57	8.38	2.20	33.44	17.72
36–45	21.87	15.04	14.63	5.60	66.44	39.94
≥46	37.62	13.81	13.25	3.15	58.11	37.33
*p*-value	0.071		0.092		0.051	
**Education**						
None	5.50	6.74	1.13	0.35	24.56	12.78
Primary	30.62	72.10	8.25	1.83	106.00	60.28
Secondary	32.62	18.78	25.25	8.46	59.11	24.04
Tertiary	3.00	0.00	2.88	0.35	3.00	0.00
*p*-value	0.991		**0.048**		0.063	
**Marital Status**						
Married	54.87	16.97	25.00	8.29	155.56	86.86
Single	7.88	4.15	5.38	1.40	13.44	3.35
Divorced	3.88	6.17	4.13	2.80	18.22	5.33
Widowed	5.13	0.64	3.13	2.03	5.44	0.882
*p*-value	**0.031**		**0.044**		**0.026**	
**Herd size**						
<25	62.13	16.63	174.22	96.10	23.50	8.142
≥25	9.00	4.92	18.44	2.12	14.63	3.739
*p*-value	**0.011**		**0.001**		**0.047**	
**District**						
CP	3.88	10.96	0.00	0.00	16.89	10.99
KA	5.00	11.31	1.00	0.00	21.33	12.63
SA	4.50	12.72	0.00	0.00	28.44	19.61
MH	4.75	13.43	0.00	0.00	23.44	14.03
CZ	25.13	3.52	16.87	4.91	23.67	4.58
TO	16.13	11.51	16.12	4.91	26.11	8.16
CK	6.00	16.97	0.00	0.00	21.11	12.53
NE	6.48	18.03	0.00	0.00	31.67	20.35
*p*-value	0.721		0.994		0.898	
Overall	17.90	12.78	9.40	2.55	41.23	22.11
Range	3.00–80.50		0.00–270.32		3.00–242.42	

Std. Deviation = Standard Deviation, boldface indicates statistical significance at *p* < 0.05.

**Table 6:** Summary of univariate regression analysis of potential predictors within the knowledge category and the observed level of knowledge for RVF.

Factors under Knowledge	Number of Participants (*n* = 400)	Knowledgeable (*n* = 33)	Proportion (%)	OR	95% CI	*p*-Value
Months for occurrence of abortions (*n* = 400) ***						
Jan, Feb, Mar	349	26	7.45	Ref		
Apr, May, Jun	51	7	13.73	3.531	1.40–8.90	0.008
What causes abortion (*n* = 400) *						
Diseases	313	21	6.71	Ref		
Poor feeding	48	9	18.75	1.159	0.32–4.07	0.818
Misfortune	39	3	7.69	3.209	1.37–7.50	0.007
Do you know what causes RVF (*n* = 400) ***						
No	367	29	7.90	Ref		
Yes	33	4	12.12	3.531	1.40–8.90	0.008
Do you know RVF clinical signs (*n* = 400)						
No	365	27	7.40	Ref		
Yes	35	6	17.14	2.590	1.98–6.78	0.053
Do you know the affected species (*n* = 400) ***						
No	362	31	8.56	Ref		
Yes	38	2	5.26	0.216	0.07–0.62	0.005
Do you know how it is transmitted (*n* = 400) ***						
No	362	24	6.63	Ref		
Yes	38	9	23.68	4.371	1.85–10.27	0.001
Can mosquito transmit RVF (*n* = 400) ***						
No	362	23	6.35	Ref		
Yes	38	10	26.32	5.264	2.28–12.15	<0.001
Do you know that it’s zoonoses (*n* = 400) ***						
No	362	23	6.35	Ref		
Yes	38	10	26.32	5.264	2.28–12.15	<0.001
Can you suspect RVF cases in livestock (*n* = 400) ***						
No	362	23	6.35	Ref		
Yes	38	10	26.32	5.264	2.28–12.15	<0.001

*n* = Number of participants; CI = Confidence interval, Significant level < 0.05; OR = Odds ratio; *** = Significant at 0.05, considered for multivariate analysis; * = considered for multivariate analysis (cut-off *p* ≤ 0.250); Ref = reference.

**Table 7:** Summary of univariate regression analysis of knowledge of RVF and potential predictors of management practices.

Factors under Practices	Number of Participants (*n* = 400)	Knowledgeable (*n* = 33)	Proportion (%)	OR	95% CI	*p*-Value
Age (*n* = 400) *						
≥ 46	159	12	7.55	Ref		
36–45	142	15	10.56	2.228	1.60–8.14	0.022
26–35	54	4	7.41	0.484	0.06–3.71	0.486
18–25	44	2	4.55	0.000	0.00–0.00	1.000
Gender (*n* = 400) ***						
Female	269	18	6.69	Ref		
Male	131	15	11.45	1.808	1.00–3.70	0.108
Education (*n* = 400) *						
None	42	7	16.67	Ref		
Primary	270	23	8.52	0.393	0.01–0.74	0.826
Secondary	85	3	3.53	0.100	0.00–0.42	0.038
Tertiary	3	0	0.00	5.370	0.0.00–0.00	0.177
Did you experience neonatal death (*n* = 400) ***						
No	61	33	54.10	Ref		
Yes	339	0	0.00	0.350	0.17–0.72	0.004
Mode of night shelter (*n* = 400) *						
Private	177	7	3.95	Ref		
Communal	216	26	12.04	3.323	1.41–7.85	0.006
None	7	0	0.00	0.000	0.00–0.00	0.999
Type of grazing grounds (*n* = 400) ***						
Communal	224	7	3.13	Ref		
Stall feeding	176	26	14.77	2.583	1.25–5.36	0.011
Herd composition (*n* = 400) ***						
Mixed species	293	13	4.44	Ref		
Single species	107	20	18.69	2.855	1.39–5.88	0.004
Management of neonatal materials (*n* = 400) ***						
Unprotected	107	12	11.21	Ref		
Protected	293	21	7.17	0.350	0.17–0.72	0.004
Abortion management (*n* = 400) ***						
Not buried	222	29	13.06	Ref		
Buried	178	4	2.25	0.190	0.08–0.44	0.001
How did you handle aborted materials (*n* = 400) ***						
Protected	221	29	13.12	Ref		
Unprotected	179	4	2.23	0.321	0.15–0.70	0.004
Can you prevent spread of RVF? (*n* = 400) ***						
Yes	38	3	7.89	Ref		
No	362	30	8.29	5.264	2.28–12.15	0.001

*n* = Number of participants; CI = Confidence interval, Significant level < 0.05; OR = Odds ratio; *** = Significant at 0.05, considered for multivariate analysis; * = considered for multivariate analysis (cut-off *p* ≤ 0.250); Ref = reference.

**Table 8:** Summary of maximum-likelihood estimates for predictors associated with RVF knowledge.

Variable	Level	aOR	95% CI	*p*-Value
What causes abortion (*n* = 400)	Diseases	Ref		
	Poor feeding	1.879	0.47–7.52	0.372
	Misfortune	3.861	1.14–13.05	0.001 ***
How RVF is transmitted (*n* = 400)	Yes	Ref		
	No	5.652	1.76–18.12	0.004 ***
Do you know the affected species (*n* = 400)	No	Ref		
	Yes	0.140	0.03–0.62	0.009 ***

*** = Significant at 0.05; aOR = adjusted Odds ratio; CI = Confidence interval; Significant at *p* < 0.05; Ref = Reference category.

## Data Availability

All materials supporting this study are included in the manuscript. The datasets analyzed during the current study are available from the corresponding author on reasonable request.
